# Paradox of Low CA-125 in Patients with Decompensated Congestive Heart Failure

**DOI:** 10.3390/biomedicines13071679

**Published:** 2025-07-09

**Authors:** Raquel López-Vilella, Borja Guerrero Cervera, Víctor Donoso Trenado, Julia Martínez-Solé, Sara Huélamo Montoro, Valero Soriano Alfonso, Franco Appiani, Luis Martínez Dolz, Luis Almenar-Bonet

**Affiliations:** 1 Unit of Heart Failure and Transplant, Hospital Universitari i Politècnic La Fe, 46026 Valencia, Spain; cune10@hotmail.com (R.L.-V.); vdonoso@outlook.com (V.D.T.); juliamsole@gmail.com (J.M.-S.); lualmenar@gmail.com (L.A.-B.); 2 Cardiology Department, Hospital Universitari i Politècnic La Fe, 46026 Valencia, Spain; huelamo_sar@gva.es (S.H.M.); valero1996@gmail.com (V.S.A.); fappiani@gmail.com (F.A.); luismartinezdolz@gmail.com (L.M.D.); 3 Centro de Investigación Biomédica en Red de Enfermedades Cardiovasculares (CIBERCV), Instituto de Salud C arlos III, 28029 Madrid, Spain

**Keywords:** heart failure, systemic congestion, CA-125, clinical profiles, survival

## Abstract

**Background/Objectives**: Patients diagnosed with decompensated congestive heart failure (HF) often have elevated CA-125 levels, attributed to systemic congestion. However, a subgroup of patients presents with normal CA-125 levels. The primary objective of this study was to characterize the clinical, analytical, and echocardiographic profiles of patients admitted for decompensated congestive HF according to their CA-125 levels. The secondary objective was to analyze mortality after discharge. **Methods**: We conducted a retrospective study of patients hospitalized for a decompensated congestive HF episode. Recruitment was consecutive over more than 4 years (December 2019–June 2024), with 3151 patients recruited. Scheduled admissions, transfers from other hospitals, pulmonary congestion patterns, mixed patterns, and low output were the exclusion criteria. The final number of patients included was 166, all with an isolated systemic congestion pattern: CA-125 ≤ 50 U/mL: 38, and CA-125 > 50 U/mL: 128. **Results**: The comparative analysis between the groups showed that patients with CA-125 ≤ 50 U/mL were more often women (*p* < 0.05). They also had lower bilirubin and GOT/AST levels (*p* < 0.05). The percentage of patients with a preserved left ventricular ejection fraction (≥50%) was higher in the CA-125 ≤ 50 U/mL group (*p* < 0.05). The right ventricular (RV) size and inferior vena cava (IVC) were enlarged in both groups but with no significant differences (*p* < 0.05). However, the degree of RV dysfunction was greater in the CA-125 > 50 U/mL group, while the proportion of patients with inspiratory collapse of the IVC was higher in the CA-125 ≤ 50 U/mL group (*p* < 0.05). Survival curves differed from the first month and throughout the follow-up, with higher mortality in the CA-125 > 50 U/mL group. Thus, the probability of being alive at the end of the follow-up was over 50% in the CA-125 ≤ 50 U/mL group, while in the CA-125 > 50 U/mL group, it was around 25% (*p* < 0.05). **Conclusions**: The proportion of patients with decompensated congestive HF and systemic congestion who present with a low CA-125 level is close to 25%. These patients are mostly women with a preserved ejection fraction and inspiratory collapse of the IVC of >50%. Moreover, they have a higher survival rate, so a low CA-125 could help identify a subgroup of patients with a better prognosis.

## 1. Introduction

Heart failure (HF) is a global disease that reduces both the life expectancy and quality of life of patients who suffer from it [1]. It generally follows a chronic course, with exacerbations that occur more or less frequently, necessitating visits to the emergency room and/or hospitalization, which, in turn, increases morbidity and mortality [2]. Among hospitalized patients with acute heart failure, there are different clinical congestion profiles, each with varying prognoses depending on the hemodynamic situation and the location of the congestion [3]. Among these profiles, studies show that the clinical picture of decompensated systemic congestion, although not the most frequent, has the worst prognosis over five years [4].

Carbohydrate antigen 125 (CA-125) is a high-molecular-weight protein synthesized in the serous membranes, primarily used as an analytical control marker for ovarian cancer, and is also elevated in patients with acute HF and congestion [5]. Its elevation depends on the degree and location of the congestion, with systemic congestion presenting the highest levels (3,4). However, even in patients hospitalized with this pattern, normal values can be found, a fact that remains unclear in the scientific literature, creating confusion in the clinical research on this novel biomarker [6,7].

The hypothesis for this study was that in patients hospitalized for decompensated HF with systemic congestion, a low CA-125 level would be associated with a specific profile that could have prognostic implications regarding survival.

This study aimed to characterize the clinical profiles of patients hospitalized for decompensated congestive HF based on their CA-125 levels. The secondary objective was to compare the mean survival times between the study groups.

## 2. Materials and Methods

**Figure 1:**
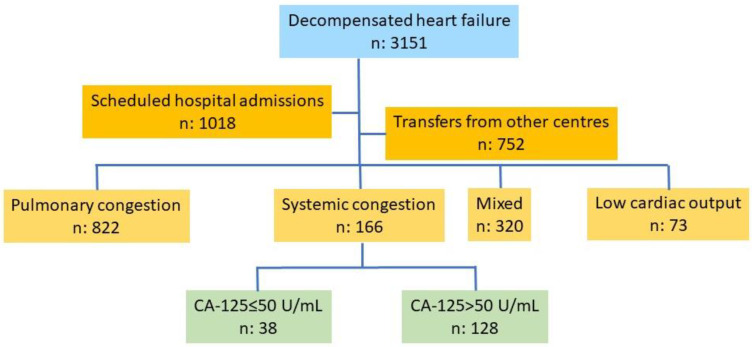
Flow chart of patient selection. Abbreviations: CA-125: carbohydrate antigen 125.

This is a retrospective study based on a database of patients consecutively admitted for an episode of decompensated HF in the Cardiology Department of a tertiary care hospital. Information was gathered during patient admission and extracted and stored by a team of cardiologists specializing in HF. Recruitment was consecutive over more than four years (December 2019–June 2024), with 3151 patients recruited. Scheduled admissions (n: 1018), transfers from other hospitals (n: 752), a pulmonary congestion pattern (n: 822), pulmonary and systemic congestion (n: 320), and a low-output pattern (n: 73) were the exclusion criteria. The final number of patients included in the study was 166, among which all had a systemic congestion pattern, 38 had normal CA-125 levels, and 128 had elevated CA-125 levels (Figure 1).

The diagnosis of acute HF with predominant decompensated systemic congestion was defined as the presence of fluid retention manifesting as a significant increase in weight, reduced diuresis, and severe edema, without evidence of orthopnea or signs of low cardiac output. Furthermore, the absence of pulmonary congestion was required, confirmed by the absence of crackles on lung auscultation and no signs of congestion on chest X-ray. In cases of doubt, patients were evaluated by a multidisciplinary team to ensure correct classification according to the established criteria.

CA-125 was considered normal up to 50 U/mL. The maximum normal value is not defined in HF. For ovarian cancer suspicion, this limit is usually 35 U/mL, although it may vary by laboratory [6,8].

The variables of interest included clinical (medical history and baseline characteristics), echocardiographic (functional assessment of both ventricles and the inferior vena cava), therapeutic (medications at the time of admission), and analytical (standard parameters for these patients based on a pre-configured analytical profile for decompensated HF) variables [9,10]. Survival time was also analyzed in both study groups.

The study was conducted in accordance with the Declaration of Helsinki. The research project was approved by the Biomedical Research Ethics Committee of La Fe University and Polytechnic Hospital, Valencia (Registration No.: 2024-0925-1, Protocol Code/Acronym: HF-125, approval date 20/11/2024).

### Statistical Analysis

Categorical variables are expressed as numbers and percentages, and numerical variables as means and standard deviations or medians and interquartile ranges, depending on the normality distribution (Kolmogorov–Smirnov test).

Comparisons between groups for qualitative variables were performed using the Pearson Chi-squared test with exact bilateral significance, and corrected with Fisher’s exact statistic when any of the expected frequencies in the 2 × 2 table were less than 5. For comparisons of quantitative variables, Student’s t-test or a Mann–Whitney U test was used for non-normal distributions of independent variables.

For Kaplan–Meier survival curves, comparisons were made using the Log Rank method. Mortality tables were used to analyze the number and percentage of deaths within 365 days by study group. The Wilcoxon statistic (Gehan) was used for statistical tests.

A *p*-value of <0.05 was considered statistically significant. The statistical software used included IBM SPSS Statistics Version 27^®®^ and Stata^®®^ Statistics/Data Analysis 16.1. Graphs were created using Power Point version 2505 and SPSS version 27.

## 3. Results

### 3.1. Clinical Data

The comparative analysis between the two groups showed that patients with CA-125 ≤ 50 U/mL included a higher proportion of women (*p* < 0.05), with some trends indicating a higher proportion of patients with a valvular etiology of HF (*p* < 0.1) (Table 1). The baseline characteristics related to functional class, cause of decompensation, number of hospital days, and length of stay were similar between the groups (*p* > 0.05) (Table 2). The percentage of patients receiving cardioactive drugs upon admission was similar between groups. The mean number of diuretics prescribed was 1.3 ± 0.9. Loop diuretics were the most commonly prescribed at admission (>80%), followed by thiazides (>30%). There were no statistically significant differences between any pharmacological groups (*p* > 0.05) (Table 3).

### 3.2. Analytical and Echocardiographic Data

When comparing analytical parameters upon admission between the two groups, few significant differences were found. Bilirubin and aspartate aminotransferase (GOT/AST) levels were lower in the low-CA-125 group (*p* < 0.05). The median CA-125 level in the study group was 23 with an interquartile range of 20, while in the CA-125 > 50 U/mL group, it was 209 with an interquartile range of 275 (*p*: 0.0001) (Table 4).

Significant differences were found in some echocardiographic parameters between the study groups. The percentage of patients with a preserved left ventricular ejection fraction (LVEF) (≥50%) was higher in the normal-CA-125 group. Right ventricular (RV) size was increased in both groups but without significant differences (*p* > 0.05). However, right ventricular dysfunction was more common in the CA-125 > 50 U/mL group (*p* < 0.05). Inferior vena cava (IVC) dilation was present in both groups, but more patients in the normal-CA-125 group showed greater than 50% inspiratory collapse (*p* < 0.05) (Table 5).

### 3.3. Survival

Survival analysis showed high mortality rates among these patients from admission and throughout the follow-up period of more than four years. The survival probability differed starting from the first month after hospitalization, with a significant trend towards higher mortality in the CA-125 > 50 U/mL group (0.07). After the first month, the differences were consistently significant (*p* < 0.05) (Table 6).

**Figure 2:**
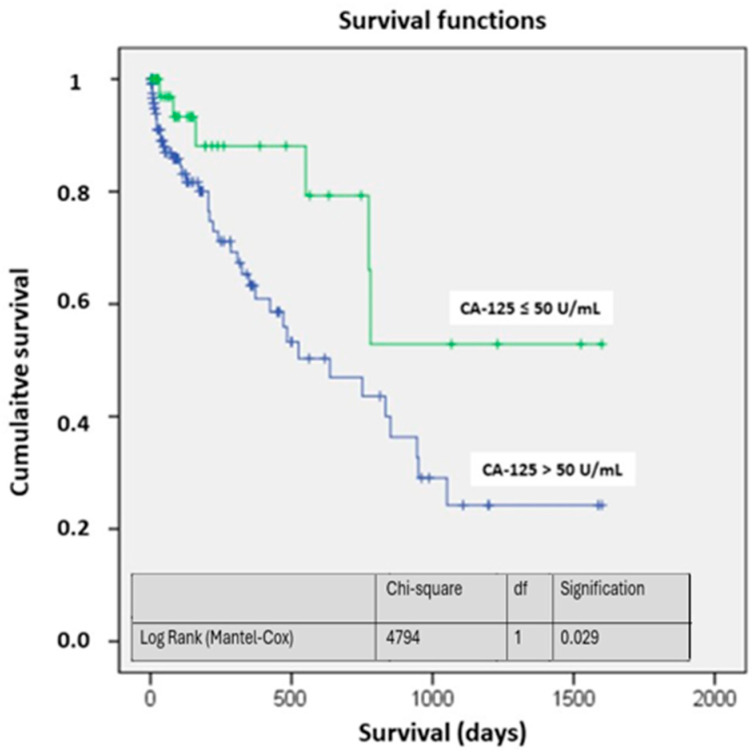
Survival based on CA-125. Abbreviations: CA-125: carbohydrate antigen 125; df: degrees of freedom.

**Figure 3:**
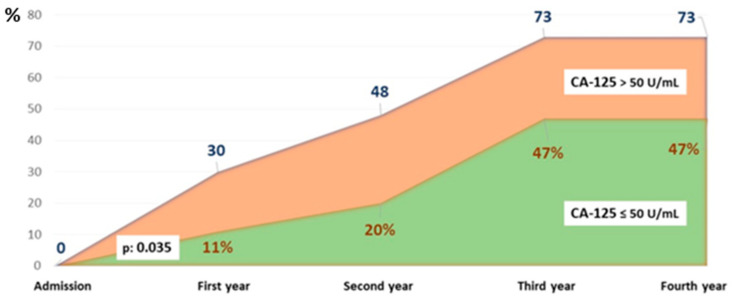
Cumulative percentage of patients who died by group and year of follow-up. Abbreviations: CA-125: carbohydrate antigen 125.

The survival curves showed significant differences, particularly at four years of follow-up. The probability of being alive in the study group was above 50%, while in the CA-125 > 50 U/mL group, it was around 25% (*p* < 0.05) (Figure 2). Figure 3 illustrates the different mortality proportions between the two groups from hospitalization and throughout follow-up, showing that the elevated-CA-125 group had higher mortality at all stages analyzed (*p* < 0.05). We have included a Graphical Abstract summarizing the results.

## 4. Discussion

CA-125 is a novel biomarker that has been linked to congestion. Of the various congestion patterns described [3], the one associated with higher CA-125 values is systemic congestion [4,5,11]. However, there is a group of patients who do not develop this elevation, which remains unexplained in the current scientific literature [6,7,12]. The aim of this study was to analyze patients admitted consecutively for decompensated congestive HF to compare whether a CA-125 ≤ 50 U/mL is associated with certain clinical characteristics and echocardiographic findings and whether it has implications for survival. It has been shown that normal CA-125 levels are more common in women with a preserved left ventricular ejection fraction (LVEF), a right ventricle (RV) with normal function or mild dysfunction, and the presence of inspiratory collapse of > 50% in the inferior vena cava (IVC) [6,7,12]. Additionally, it has a clear prognostic relationship, so it could enable the identification of a subgroup of patients with a better prognosis.

Regarding the chosen cutoff point (50 U/mL), it should be noted that the maximum normal value is not defined in heart failure, with the value commonly used in ovarian cancer being 35 U/mL [8,13]. Only one study analyzed cutoff points in acute HF (<23 U/mL); however, this cutoff was established based on a retrospective cohort analysis with a validation cohort including patients only up to 2018, and the timing of CA-125 measurement differed slightly between the two cohorts [14]. The 50 U/mL threshold helps reduce variability by excluding smaller elevations that may not be clinically significant, ensuring that only cases with a relevant increase in CA-125 are considered. In this sense, standardizing and homogenizing the diagnostic tests performed on patients admitted for HF decompensation would contribute to establishing better knowledge of this molecule and improved clinical management of patients, as CA-125 is still not routinely requested in many centers [9,10]. To achieve this, the use of pre-configured analytical profiles that systematically include this biomarker is essential [10].

In this study, 23% of the patients had low CA-125 levels despite being admitted for acute HF with clear systemic congestion. There are factors that have not been clarified, which explain why normal CA-125 values are found in populations that typically present elevated levels. In the BIOSTAT-CHF cohort (1583 patients with acute congestive heart failure), 34.6% of the patients had CA-125 levels below 35 U/mL [15]. Even in women with advanced serous ovarian cancer, which represents the paradigm of elevated CA-125, some series report up to 10–20% of patients with normal levels [16,17].

In our series, patients admitted for acute HF with low CA-125 levels at admission were more frequently women. Other baseline characteristics showed no significant differences between the groups. In other published series, the female sex has also been described as an independent predictor of low CA-125 [6]. Atrial fibrillation (AF) and its recurrence after ablation have been linked to elevated plasma levels [18,19], although we did not find this relationship in this study of patients with acute congestive HF.

Regarding pharmacological treatment, up to one-third of patients in our series were receiving treatment with three diuretic drugs, with sequential nephron blockade being the usual strategy employed in congestive patients [3]. Many patients with reduced-ejection-fraction heart failure (HFrEF) are already treated with mineralocorticoid receptor antagonists as part of HF treatment [20]. The low number of patients treated with sodium–glucose cotransporter type 2 inhibitors (SGLT2is) is notable, but it should be considered that patient recruitment started in 2019 when their level of recommendation in heart failure guidelines was not yet established. One study evaluated the effect of dapagliflozin on CA-125 levels in the short term in chronic HFrEF, finding that this drug was associated with a significant reduction in CA-125 [21]; however, this has not been analyzed in acute HF, for which the literature is scarce.

From an analytical perspective, it is common for patients with congestive heart failure to have renal impairment and develop cardiorenal syndrome [22,23,24]. In this series, no differences were found in renal function, ions, hemograms, or amino-terminal fragment of the brain natriuretic peptide (NT-proBNP). The absence of a relationship between CA-125 and renal function is an expected finding, as it has been demonstrated that this biomarker is not affected by renal function [25]. In contrast, some studies have found a direct association between CA-125 and NT-proBNP levels [6,7]. Patients with low CA-125 had lower levels of bilirubin and liver enzymes (AST). Since bilirubin and transaminases are especially elevated in right ventricular failure, it is justified that higher levels of these biomarkers are associated with elevated CA-125 levels [4,26]. Bilirubin and AST are primarily elevated in response to hepatic congestion and hypoperfusion, while alanine aminotransferase (ALT) is more specific for pure hepatocellular damage and less sensitive to hypoxia [27]. One possible hypothesis for the lack of significant differences in NT-proBNP levels between groups is that both groups had a similarly high baseline risk and comparable degrees of congestion at admission. Lower AST and bilirubin levels are associated with less systemic congestion and better prognosis in the context of HF [28], although in the specific scenario of congestion with low CA-125, this correlation has not been specifically studied.

From an echocardiographic perspective, patients with CA-125 < 50 U/mL were more likely to have preserved LVEF, along with normal or mildly depressed RV function. Other echocardiographic parameters showed no significant differences, nor did the size of the IVC. However, in the group with CA-125 < 50 U/mL, half of the patients had inspiratory collapse of the IVC of >50%. The relationship between CA-125 levels and echocardiographic parameters has shown contradictory results in some studies. In several studies in patients with congestive heart failure, CA-125 levels did not correlate with LVEF or the left ventricular end-diastolic diameter [29,30]. However, other groups described a positive correlation between CA-125 levels and pulmonary systolic pressure and a negative correlation with the ejection fraction [31]. The size of the IVC, part of the VEXUS (Venous Excess Ultrasound) score, was also not associated with higher CA-125 levels in the present study. It is important to remember that VEXUS is not yet validated in all settings and may be affected by frequent alterations in patients with HF, such as the degree of tricuspid regurgitation [23,24]. Regarding the collapse of the IVC, there are no studies that analyze the collapse and its size separately with CA-125. However, it has been described that there is a greater (inverse) correlation between the percentage of IVC collapse and the degree of venous congestion (measured using central venous pressure (CVP)), compared to the direct correlation between CVP and IVC diameter [32,33].

As for survival, the curves differ since admission, and the probability of survival shows significant differences at one year, which become more pronounced at 3–4 years. Other observational studies analyzing two-year survival based on CA-125 and NT-proBNP levels in acute HF have also reproduced these results, with CA-125 being an independent prognostic factor for survival, and with higher levels being associated with higher mortality [34]. Mansour IN et al. analyzed survival according to CA-125 levels at admission, divided into quartiles, in decompensated heart failure. Patients in the highest quartile with CA-125 levels >50.6 U/mL had a 46% mortality rate at 40 months, compared to the group with levels below 8.3 U/mL, which had a mortality rate of 13.6% [35].

The high 4-year mortality in the group with congestive HF and elevated CA-125 levels is striking. This group has been shown to have the worst prognosis in some series, even worse than patients with low-output heart failure [4,36]. This could be justified because patients with systemic congestion more often have poorer renal function and RV function, both independent predictors of poorer survival [37].

Thus, CA-125 is a recently used clinical biomarker in HF, associated with congestion and with prognostic value, especially in a chronic outpatient context [38]. However, it is a biomarker that is not heart-specific and can be elevated in oncological or inflammatory diseases [3,5], and a significant percentage of patients, nearly 25% in our series, may present normal values of this biomarker despite congestive heart failure decompensation. Furthermore, its cutoff point is not fully validated in HF, with the value commonly used being that for ovarian cancer (35 U/mL) [8,14], and the cutoff points in HF come from retrospective cohorts [15]. It still does not have the same validity as other biomarkers used in cardiology, such as troponins or NT-proBNP. NT-proBNP is widely validated, organ-specific, and part of the heart failure diagnostic algorithm in clinical practice guidelines [22]. Therefore, in the context of acute HF, CA-125 should still be considered an experimental biological marker, and more basic and clinical studies are needed to help standardize it. Future studies should aim to better understand the interaction between systemic inflammation, venous congestion, and serosal activation, which might explain CA-125 fluctuations.

This is a retrospective, single-center study based on a database compiled upon patient discharge. Other causes that might elevate this biomarker were not analyzed, although in some cases, a gynecologist was consulted to perform tumor screening tests, all of which were negative. A value of 50 was chosen to balance the groups, as there is no recommended value in the literature specifically for heart failure, and the values established are related to ovarian neoplasia. On the other hand, a value of 50 helps avoid giving importance to minor elevations without clinical significance. CA125 was not assessed at discharge despite the average hospital stay being nearly 10 days. However, the series is consecutive, which lends reliability to the results, and was completed by the team of cardiologists in the Heart Failure Unit. Additionally, all data were verified by a single cardiologist, who is also part of the team, before inclusion in the database.

## 5. Conclusions

Based on this study’s results, it can be concluded that the relationship of patients with HF and decompensated systemic congestion requiring hospitalization and presenting low CA-125 is 1:4. These patients are mostly women with preserved LVEF, normal or slightly reduced RV function, and inspiratory collapse of the inferior vena cava of >50%. Moreover, they have a better survival rate, so a low CA-125 at admission in this cohort of patients could identify a subgroup with a better prognostic evolution.

## Figures and Tables

**Table 1:** Baseline characteristics of patients.

	CA-125 ≤ 50 U/mL n: 38	CA-125 > 50 U/mL n: 128	*p*	Total n: 166
Age (years) #	70 ± 11	69 ± 12	0.683	70 ± 12
Sex (male)	16 (42)	94 (73)	0.001	110 (66)
Baseline heart disease:				
HT	6 (16)	16 (13)	0.599	22 (13)
Ischemic	8 (21)	37 (29)	0.339	45 (27)
IDCM	3 (8)	14 (11)	0.811	17 (10)
Valvular	16 (42)	35 (27)	0.083	51 (31)
Other	5 (13)	26 (20)	0.320	31 (19)
Previous CVS	10 (26)	36 (28)	0.807	46 (28)
HT	32 (84)	93 (73)	0.199	125 (75)
Dyslipidemia	18 (47)	79 (62)	0.135	97 (58)
Diabetes mellitus	14 (38)	55 (43)	0.706	69 (42)
Active smoking *	14 (38)	59 (47)	0.354	73 (44)
Active drinking	3 (8)	6 (5)	0.428	9 (6)
COPD	2 (5)	14 (11)	0.367	16 (10)
SAHS	5 (13)	13 (16)	0.537	18 (11)
Obesity	7 (18)	22 (17)	0.860	29 (17)
Renal failure	16 (42)	55 (43)	0.994	71 (43)
Hypothyroidism	5 (13)	11 (10)	0.533	16 (10)
Atrial fibrillation	22 (58)	82 (65)	0.452	104 (63)
Stroke	2 (5)	14 (11)	0.352	16 (10)
PVD	2 (5)	6 (5)	0.898	8 (7)
Peritoneal dialysis	1 (3)	3 (2)	0.929	4 (4)

# Kolmogorov–Smirnov < 0.05. Median and interquartile range. The rest of the values are expressed as absolute numbers and percentages (in parentheses). * Active smoker or ex-smoker <1 year. Abbreviations: CA-125: carbohydrate antigen 125; COPD: chronic obstructive pulmonary disease; CVS: cardiovascular surgery; HT: hypertension; IDCM: idiopathic dilated cardiomyopathy; PVD: peripheral vascular disease; SAHS: sleep apnea–hypopnea syndrome.

**Table 2:** Clinical profiles of patients.

	CA-125 ≤ 50 U/mL n: 38	CA-125 > 50 U/m n: 128	*p*	Total n: 166
De novo HF	4 (11)	19 (15)	0.601	13 (14)
Functional class (NYHA)			0.251	
I	1 (3)	7 (5)		8 (5)
II	25 (65)	65 (51)		90 (54)
III, IV	12 (32)	56 (44)		68 (41)
Cause of decompensation			0.707	
Disease progression	21 (55)	76 (59)		97 (58)
Infections	4 (11)	11 (9)		15 (9)
Arrhythmias	6 (16)	14 (11)		20 (12)
Unknown	2 (5)	8 (6)		10 (6)
Other	5 (13)	19 (15)		24 (15)
Number of previous admissions #	2.7 ± 2.2	2.7 ± 2.6	0.972	2.7 ± 2.5
Days of admission #	9.6 ± 6.4	10.0 ± 9.1	0.822	9.9 ± 8.5
Size (cm) #	164 ± 8	163 ± 11	0.390	163 ± 10
Weight (Kg) #	78 ± 17	80 ± 21	0.401	80 ± 20
SBP (mmHg) #	123 ± 26	125 ± 25	0.628	125 ± 25
DBP (mmHg) #	69 ± 13	75 ± 21	0.093	74 ± 19
Heart rate (bpm) #	81 ± 12	85 ± 24	0.325	84 ± 21

All values are those at admission. # Kolmogorov–Smirnov > 0.05. Mean and standard deviation. The rest of the values are expressed as absolute numbers and percentages (in parentheses). Abbreviations: CA-125: carbohydrate antigen 125; DBP: diastolic blood pressure; HF: heart failure; NYHA: New York Heart Association; SBP: systolic blood pressure.

**Table 3:** Treatment prior to admission.

	CA-125 ≤ 50 U/mL n: 38	CA-125 > 50 U/mL n: 128	*p*	Total n: 166
ACEI/ARB/ARNI	19 (50)	54 (42)	0.458	73 (44)
Beta-blockers	21 (55)	76 (59)	0.669	97 (58)
MRA	17 (46)	64 (50)	0.711	81 (49)
SGLT2i	5 (13)	33 (26)	0.126	38 (23)
Loop diuretic	31 (82)	108 (84)	0.802	139 (84)
Thiazides	10 (27)	43 (34)	0.550	53 (32)
Tolvaptan	2 (5)	12 (9)	0.738	14 (8)
Acetazolamide	5 (5)	10 (8)	0.891	15 (9)
Number of diuretics #@	1.2 ± 0.8	1.4 ± 0.9	0.419	1.3 ± 0.9

# Kolmogorov–Smirnov > 0.05. Mean and standard deviation. The rest of the values are expressed as absolute numbers and percentages (in parentheses). @ Excluding ARM and iSGLT2. Abbreviations: ACEI/ARB: angiotensin-converting enzyme inhibitor/angiotensin receptor blocker; ARNI: angiotensin receptor and neprilysin inhibitor; CA-125: carbohydrate antigen 125; MRA: mineralocorticoid receptor antagonist; SGLT2i: Sodium–glucose cotransporter 2 inhibitor.

**Table 4:** Blood tests on admission.

	CA-125 ≤ 50 U/mL n: 38	CA-125 > 50 U/mL n: 128	*p*	Total n: 166
Urea (mg/dL)	66 (69)	80 (84)	0.443	73 (78)
Creatinine (mg/dL)	1.6 (1.5)	1.6 (1.1)	0.426	1.6 (1.1)
Glomerular filtration rate (mL/min/1.73 m2)	38 (31)	42 (36)	0.473	42 (34)
Bilirubin (mg/dL)	0.7 (0.5)	1.2 (1.2)	0.003	1.0 (1.1)
GOT/AST (U/L)	18 (12)	25 (15)	0.037	24 (14)
GPT/ALT (U/L)	14 (10)	18 (15)	0.139	17 (14)
usTnT (ng/L)	43 (36)	58 (52)	0.145	52 (44)
NT-proBNP (pg/mL)	5770 (4685)	5902 (6058)	0.193	5836 (5494)
Sodium (mEq/L)	139 (7)	136 (11)	0.160	138 (9)
Potassium (mEq/L)	4.1 (0.9)	4.0 (1.1)	0.867	4.0 (1.1)
Hemoglobin (g/dL)	11.0 (3.2)	12.9 (2.3)	0.772	12.5 (2.8)
Hematocrit (%)	34 (8)	41 (9)	0.439	39 (11)
Platelets (µL, ÷100)	168 (92)	210 (81)	0.378	201 (82)
Uric acid (mg/dL)	8.4 (3.5)	8.6 (2.7)	0.593	8.6 (2.8)
TSAT (%)	22 (12)	18 (11)	0.452	19 (12)
Ferritin (ng/mL)	136 (322)	165 (340)	0.565	161 (337)
CA-125 (U/mL)	23 (20)	209 (275)	0.0001	148 (270)

Median and interquartile range. The rest of the values are expressed as absolute numbers and percentages (in parentheses). Abbreviations: ALT (GPT), alanine aminotransferase; AST (GOT), aspartate aminotransferase; CA-125: carbohydrate antigen 125; TSAT: transferrin saturation; NT-proBNP: N-terminal pro-B-type natriuretic peptide; usTnT: ultrasensitive troponin.

**Table 5:** Echocardiographic evaluation.

	CA-125 ≤ 50 U/mL n: 38	CA-125 > 50 U/mL n: 128	*p*	Total n: 166
Preserved LVEF (≥50%)	22 (58)	51 (40)	0.038	73 (44)
Dilated LV (LV-EDD > 56 mm)	12 (32)	46 (36)	0.450	58 (35)
LVH (>12 mm)	23 (61)	64 (50)	0.170	87 (52)
Severe left atrial dilatation (≥50 mm)	14 (37)	52 (41)	0.765	66 (40)
Significant valvulopathies *				
AoR	1 (3)	4 (3)	0.663	5 (3)
AoS	3 (8)	7 (5)	0.702	10 (6)
MR	7 (18)	19 (15)	0.620	26 (16)
MS	0 (0)	4 (3)	0.573	4 (2)
TR	15 (39)	44 (34)	0.565	59 (36)
RV function (TAPSE) @			0.004	
Normal	18 (46)	53 (41)		71 (43)
Mild dysfunction	13 (36)	17 (14)		30 (18)
Moderate dysfunction	5 (13)	31 (24)		36 (22)
Severe dysfunction	2 (5)	27 (21)		29 (17)
Dilated RV (basal diameter > 40 mm)	27 (71)	83 (65)	0.471	110 (66)
Inferior vena cava (mm) #	23 (7)	23 (4)	0.085	23 (5)
Vena cava collapse ≥ 50%	20 (53)	23 (18)	0.001	43 (26)
PH (PAsP ≥ 50 mmHg)	19 (50)	88 (69)	0.102	107 (64)

Median and interquartile range. The rest of the values are expressed as absolute numbers and percentages (in parentheses). * moderate–severe + severe. @ TAPSE intervals: Normal: TAPSE ≥ 17 mm; Mild dysfunction: TAPSE 13–16 mm; Moderate dysfunction: TAPSE 10–12 mm; Severe dysfunction: TAPSE < 10 mm. Abbreviations: AoS: aortic stenosis; AoR: aortic regurgitation; CA-125: carbohydrate antigen 125; LV-EDD: left ventricular end-diastolic diameter; LVEF: left ventricular ejection fraction; MS: mitral stenosis; MR: mitral regurgitation; PH: pulmonary hypertension; LVH: left ventricular hypertrophy; PAsP: pulmonary artery systolic pressure; RV: right ventricle; TAPSE: tricuspid annular plane systolic excursion; TR: tricuspid regurgitation. # Kolmogorov–Smirnov < 0.05.

**Table 6:** Survival probability at follow-up.

	CA-125 ≤ 50 U/mL n: 38	CA-125 > 50 U/mL n: 128	*p*	Total n: 166
30 days	97	91	0.074	93
1 year	88	63	0.0001	69
2 years	79	47	0.0001	54
3 years	53	24	0.0001	31
4 years	53	24	0.0001	31

The values represent survival percentages. Abbreviations: CA-125: carbohydrate antigen 125.

## Data Availability

The dataset is available upon request to the authors.

## Abbreviations

The following abbreviations are used in this manuscript:

ALT	Alanine aminotransferase
CA-125	Carbohydrate antigen 125
CVP	Central venous pressure
GOT/AST	Aspartate aminotransferase
HF	Heart failure
HFrEF	Heart failure with reduced ejection fraction
IVC	Inferior vena cava
LVEF	Left ventricular ejection fraction
NTproBNP	Amino-terminal fragment of the brain natriuretic peptide
RV	Right ventricle
SGLT2i	Sodium–glucose cotransporter type 2 inhibitor
VEXUS	Venous Excess Ultrasound
